# Plasmablastic Transformation of CLL/SLL: The Role of Early NGS Diagnosis and Targeted Multimodal Therapy

**DOI:** 10.3390/diagnostics16050702

**Published:** 2026-02-27

**Authors:** Jelena Filipović, Sara Milošević, Tatjana Terzić, Thorsten Braun, Ramy Rahmé, Grégory Lazarian, Thami Benboubker, Michael Soussan, Antoine Martin

**Affiliations:** 1 Institute of Pathology, Faculty of Medicine, University of Belgrade, 11000 Belgrade, Serbia; vjesticaj@gmail.com (J.F.); tatjanaterzic63@gmail.com (T.T.); 2 Faculty of Medicine, University of Belgrade, 11000 Belgrade, Serbia; sara020501@gmail.com; 3 Department of Hematology, Université Paris Nord, Assistance Publique–Hôpitaux de Paris (AP-HP), Hôpital Avicenne, 93000 Bobigny, France; thorsten.braun@aphp.fr (T.B.); ramy.rahme@aphp.fr (R.R.); 4 Biological Hematology Department, Assistance Publique–Hôpitaux de Paris (AP-HP), Hôpital Avicenne, 93000 Bobigny, France; gregory.lazarian@aphp.fr (G.L.); thami.benboubker@aphp.fr (T.B.); 5 Department of Nuclear Medicine, Université Paris Nord, Assistance Publique–Hôpitaux de Paris (AP-HP), Hôpital Avicenne, 93000 Bobigny, France; michael.soussan@aphp.fr; 6 Department of Anatomical and Cytological Pathology, Université Paris Nord, Assistance Publique–Hôpitaux de Paris (AP-HP), Hôpital Avicenne, 93000 Bobigny, France

**Keywords:** SLL, PBL, NGS-based clonal confirmation, CD38 and BTK-targeted therapy

## Abstract

**Background and Clinical Significance:** Plasmablastic lymphoma (PBL) is a rare and highly aggressive B-cell neoplasm most often associated with immunodeficiency. Transformation of chronic lymphocytic leukemia/small lymphocytic lymphoma (CLL/SLL) into PBL is exceptionally uncommon, particularly in immunocompetent individuals. This paper describes a rare synchronous SLL-to-PBL transformation and summarizes current knowledge on synchronous and metachronous cases reported in the literature. **Case Presentation** A midle-aged immunocompetent patent presented with generalized lymphadenopathy and lumbar pain. Concurrent biopsies of an axillary lymph node and a retroperitoneal mass were obtained. Diagnostic evaluation included immunohistochemistry; fluorescent in situ hybridization (FISH); PCR-based assessment of IGH, IGK, and IGL loci; and next-generation sequencing (NGS) of IGHV to assess clonal relatedness. The patient was treated with six cycles of Dara-CHOP, followed by autologous stem cell transplantation and maintenance therapy with daratumumab and ibrutinib. The axillary node showed SLL (CD20+, CD5+, CD23+), while the retroperitoneal mass demonstrated classic features of PBL (CD138+, MUM1+, MYC+, Ki-67 ~100%, CD20−). FISH detected MYC rearrangement in the PBL component. PCR and NGS confirmed identical IGHV1-69 rearrangements, establishing clonal relatedness and Richter transformation. A review of published cases shows that both synchronous and metachronous CLL/SLL-to-PBL transformations are exceedingly rare. The patient achieved partial metabolic remission after treatment and remains in sustained metabolic response 24 months after diagnosis. **Conclusions**: This case highlights a rare example of synchronous CLL/SLL-to-PBL transformation in an immunocompetent patient. Integration of detailed molecular diagnostics enabled early recognition and guided a personalized treatment approach incorporating CD38-targeted therapy and BTK inhibition, resulting in an excellent long-term clinical outcome.

## 1. Introduction

Chronic lymphocytic leukemia (CLL) is the most common leukemia in Western countries, accounting for approximately 25–30% of adult leukemias. Richter’s transformation (RT) refers to the progression of CLL/small lymphocytic lymphoma (SLL) into a more aggressive lymphoma, most commonly diffuse large B-cell lymphoma (DLBCL), the most frequent subtype of non-Hodgkin lymphoma worldwide. This transformation occurs in approximately 2–10% of patients during the course of the disease and represents a clinically significant event [1,2]. Plasmablastic lymphoma (PBL) is a rare and aggressive transformation, especially uncommon in immunocompetent individuals [2]. It is most often seen in the context of immunodeficiency, such as HIV infection or prior exposure to chemotherapy [3,4,5]. We report a rare synchronous SLL and PBL case in an immunocompetent patient, and we additionally analyzed published cases of CLL/SLL-to-PBL transformations, focusing on their clinical presentation, diagnostic features, and therapeutic approaches, highlighting patterns relevant for early recognition and management.

## 2. Case Report

We report a rare case of synchronous SLL and PBL in a middle-aged patient, who presented in late 2023 with lower back pain, highlighting both diagnostic challenges and histopathological features.

18F-FDG PET/CT revealed intensely hypermetabolic retroperitoneal masses compressing the right ureter without evidence of renal impairment, along with bilateral pelvic lymph node involvement, with SUVmax values reaching up to 18.6. Shortly thereafter, biopsies of an axillary lymph node—selected for accessibility despite unreported SUVmax values—and a retroperitoneal lymph node were performed in the setting of significant lymphadenopathy on imaging. The complete blood count was normal, with an absolute lymphocyte count of 1.9 G/L. Examination of the blood smear revealed no cytological abnormalities. Immunophenotyping of peripheral blood by flow cytometry revealed a monoclonal B-cell population expressing CD5, CD23, and FMC7, with low expression of CD20 and λ light chain, and negative for CD79b. This population accounted for less 0.5 G/L of circulating B cells, consistent with a diagnosis of small lymphocytic leukemia.

Biopsy specimens were analyzed histologically and immunohistochemically.

Axillary and retroperitoneal lymph nodes were examined for morphology and immunophenotype using standard immunohistochemical panels. The antibody panel was designed to assess mature B-cell lineage, plasma cell differentiation, germinal center derivation, and relevant differential diagnostic entities, in accordance with current WHO recommendations for the diagnosis of plasmablastic lymphoma [3], and included CD20, CD5, CD23, CD10, BCL6, cyclin D1, CD138, CD38, MUM1, c-MYC, Ki-67, and immunoglobulin light chains.

FISH analysis was performed to detect MYC, BCL2, and BCL6 rearrangements. Clonal relatedness between SLL and PBL components was assessed by PCR-based analysis of IGH, IGK, and IGL loci, complemented by next-generation sequencing (NGS) of IGHV. In situ hybridization for EBV (EBER) and HHV8 (LANA1) was also performed. The patient was treated with Dara-CHOP chemotherapy (daratumumab, cyclophosphamide, doxorubicin, vincristine, and prednisone) followed by autologous hematopoietic stem cell transplantation (AHSCT). Maintenance therapy consisted of monthly daratumumab and ibrutinib.

Clinical and pathological features of reported transformation cases from CLL/SLL to PBL were analyzed separately for synchronous and metachronous transformations. Synchronous cases were defined as those in which CLL/SLL and PBL were diagnosed simultaneously or within a 6-month interval, while metachronous cases were defined as those in which transformation occurred more than 6 months after the initial CLL/SLL diagnosis. This distinction was made because synchronous transformations, occurring within a short temporal window, are more likely to originate from the same precursor clone, whereas metachronous cases may develop through later genetic divergence or therapy-driven clonal evolution, making their clonal relationship less predictable unless formally confirmed by molecular testing. For both groups, we collected and compared available clinical characteristics, pathological findings, immunophenotypic profiles, molecular data, treatment approaches, and patient outcomes, as reported in the literature.

## 3. Results

### 3.1. Morphological and Immunohistochemical Evaluation

**Figure 1:**
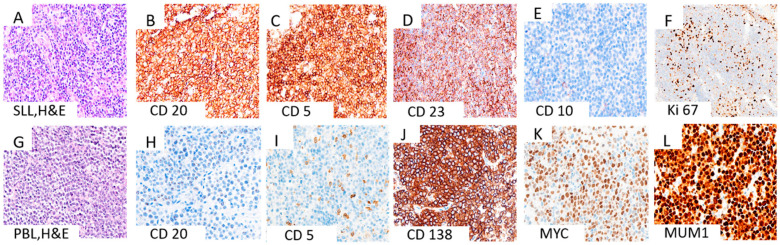
Histopathological and immunophenotypic features of SLL and its transformation into PBL. (**A**–**F**) Representative images of SLL showing: (**A**) diffuse infiltrate of small mature lymphocytes with clumped chromatin (H&E); (**B**) diffuse CD20 positivity; (**C**) co-expression of CD5; (**D**) CD23 positivity; (**E**) absence of CD10 expression; (**F**) low Ki-67 proliferation index exceeded in proliferative centers. (**G**–**L**) Corresponding images of the transformed PBL showing: (**G**) diffuse sheets of large atypical cells with plasmablastic morphology (H&E); (**H**) loss of CD20 expression; (**I**) partial expression of CD5; (**J**) strong expression of plasma cell marker CD138; (**K**) diffuse and strong nuclear expression of MYC; (**L**) diffuse nuclear positivity for MUM1. Abbreviations: SLL, small lymphocytic lymphoma; PBL, plasmablastic lymphoma; H&E, hematoxylin and eosin; magnification, 40×.

**Figure 2:**
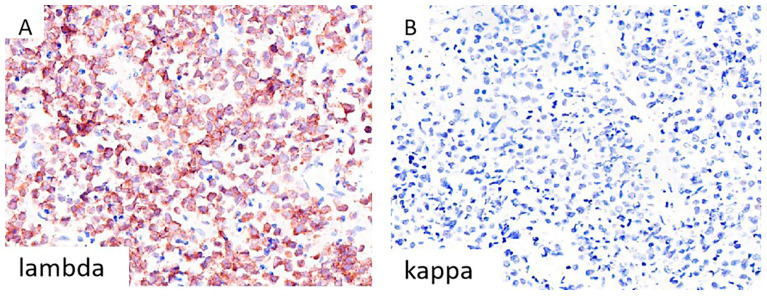
A supportive finding for the diagnosis of PBL with lambda light chain restriction. Immunohistochemically, diffuse cytoplasmic staining for lambda light chain is observed in the majority of neoplastic cells (**A**), while no kappa expression is detected (**B**). Abbreviations: PBL, plasmablastic lymphoma; magnification, 40×.

An axillary lymph node biopsy revealed diffuse infiltration by small neoplastic cells with round-to-slightly-irregular nuclei, condensed chromatin, and scant cytoplasm (Figure 1A). Immunohistochemistry confirmed SLL/CLL phenotype: CD20+, CD5+, CD23+, CD10−, BCL6−, cyclin D1−. The Ki67 proliferation index was low (20–30%), with focal enhancement in proliferative centers (Figure 1B–F). Retroperitoneal lymph node histology showed a compact lymphoid proliferation of medium-to-large plasmacytoid cells with rounded/angular nuclei, decondensed chromatin, and prominent nucleoli. Apoptotic debris and mitoses were observed. Tumor cells were CD138+, CD38+, MUM1+, c-myc+, CD5−, CD10−, CD19−, CD20−, CD23−, CD45−, BCL2−, BCL6−, EMA−, cyclin D1−, ALK1−, IgA−, OCT2−, LANA1−. The Ki67 index was nearly 100% (Figure 1G–L). Tumor cells expressed lambda light chains, but were negative for kappa (Figure 2A,B). EBV and HHV8 were negative. In situ hybridization for Epstein–Barr virus-encoded RNA (EBER) was negative in the plasmablastic lymphoma component, and immunohistochemistry showed complete loss of PAX5 expression (Supplementary Figure S1).

### 3.2. Molecular Analysis

**Figure 3:**
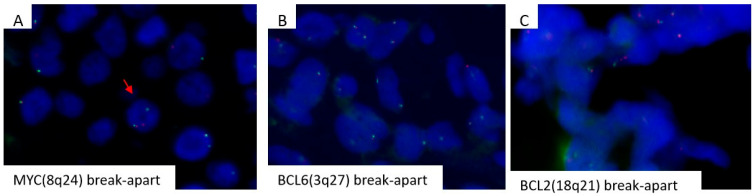
Representative FISH images using break-apart probes (MetaSystems) for MYC (8q24), BCL6 (3q27), and BCL2 (18q21). (**A**) The MYC probe demonstrates a break-apart signal pattern indicative of an MYC rearrangement (arrow). In contrast, (**B**) the BCL6 and (**C**) BCL2 probes show fused signals, consistent with the absence of BCL6 and BCL2 gene rearrangements; Abbreviation: FISH, fluorescence in situ hybridization.

**Figure 4:**
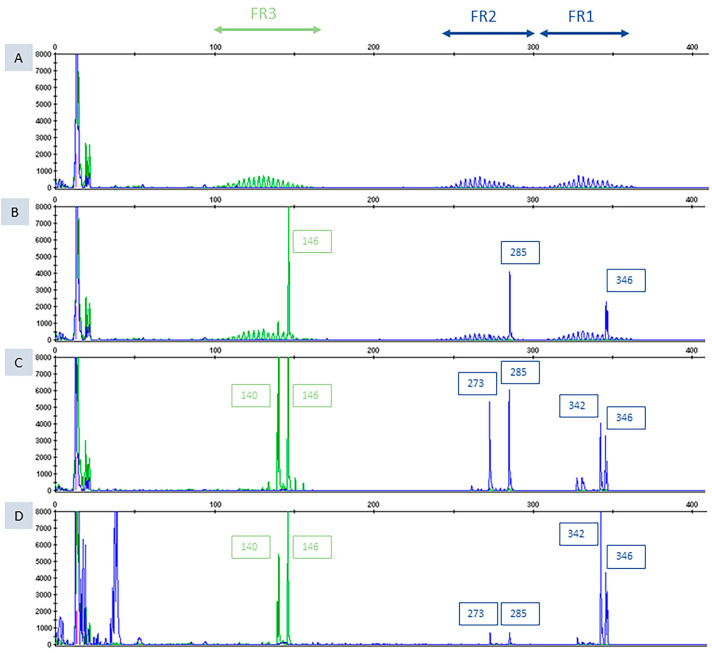
Graphical representation of B clonality (IgH locus) results. Y axis: fragments height in fluorescence intensity; X axis: fragments size in base pairs (size of the expected fragments: VH-FR1-JH 310-360; VH-FR2-JH 250-295; VH-FR3-JH 100-170). Peak sizes are shown in boxes. (**A**) Polyclonal control (Gaussian distribution of the size of the fragments amplified), (**B**) positive control (presence of a clonal rearrangement (1 peak) in the FR1, FR2 and FR3 region), (**C**) lymph-node biopsy (presence of a clonal rearrangement (2 peaks) in the FR1, FR2 and FR3 region), (**D**) retro-peritoneal biopsy (presence of the same clonal rearrangement (2 peaks of the same size) as in the lymph-node biopsy). Abbreviations: IgH, immunoglobulin heavy chain; VH, variable region of heavy chain; FR, framework region; JH, joining region of heavy chain.

FISH analysis detected MYC rearrangement in PBL; BCL2 and BCL6 translocations were absent (Figure 3A–C). PCR analysis demonstrated an identical IGHV1-69/IGHD3/IGHJ6 rearrangement in both SLL and PBL (Figure 4). This finding was further confirmed by NGS analysis, establishing clonal relatedness and supporting the diagnosis of Richter transformation.

### 3.3. Therapy

After two cycles of Dara-CHOP, follow-up PET showed no progression but did show persistent hypermetabolic lesions, prompting four additional cycles. Several months later, a complete metabolic response was observed in the abdominal mass, though pelvic lymphadenopathy remained metabolically active. Subsequently, the patient underwent AHSCT. Chemotherapy was well tolerated; aplasia occurred on day + 2, and G-CSF started on day + 7, with uneventful recovery. In July 2024, the patient underwent AHSCT as consolidation therapy. Maintenance therapy for residual SLL/CLL was subsequently initiated with daratumumab and ibrutinib.

### 3.4. Brief Review of Synchronous Versus Metachronous Transformations Reported in the Literature

The results of our literature review are presented in Table 1 and Table 2. In summary, we identified four simultaneously diagnosed transformation cases within less than six months and ten non-simultaneously diagnosed cases. In both groups, patients were predominantly elderly males, with heterogeneous clinical presentations, diagnostic approaches, and treatment modalities. CHOP-based regimens were the most frequently applied therapy. In the synchronous group, only our patient achieved clinical stability with a daratumumab-based regimen targeting CD38 in addition to prior CHOP therapy, whereas in the metachronous group, one patient responded to combined localized therapy and chemotherapy.

## 4. Discussion

PBL is a rare and aggressive B-cell neoplasm first described by Delecluse et al. in 1997, predominantly affecting older male patients and immunocompromised individuals such as those with HIV, EBV infection, or post-transplant immunosuppression [3,4,14,15]. Extranodal involvement is common, while purely nodal PBL in immunocompetent patients remains exceedingly rare [3,13,16]. Transformation of CLL/SLL into PBL is particularly uncommon. The first reported case was described by Robak et al. in 2001 in an HIV- and EBV-negative woman [17].

Early (≤6 months) versus late (>6 months) transformations may reflect distinct biological mechanisms: early transformation could result from a pre-existing aggressive sub-clone or a common progenitor at the time of CLL diagnosis, whereas late transformation may arise from clonal evolution under therapeutic or micro-environmental pressure [4,15]. Based on this hypothesis, we reviewed the published cases and stratified synchronous and metachronous transformations in Table 1 and Table 2 to highlight potential differences in pathogenesis and clinical behavior.

Precise diagnosis typically involves a lymph node biopsy, supported by ancillary studies such as IHC, FISH, PCR, and NGS to confirm clonality [2,7,11]. We report a rare case in an immunocompetent middle-aged patien, who presented with lumbar pain, bilateral renal obstruction, and generalized lymphadenopathy. SLL and PBL were diagnosed simultaneously from axillary and retroperitoneal lymph nodes. While most reported patients are older males, only a few female cases—including ours—have been described [6,12,18,19]. The clinical presentation in our patient, with generalized lymphadenopathy and lumbar pain, aligns with the most commonly reported manifestations in the literature, which often also include B symptoms and pain associated with retroperitoneal or skeletal involvement [20,21]. Unlike most reported cases, the transformation in our patient was confined to lymph nodes, with no bone marrow involvement. This localized presentation may indicate an early stage of transformation or a nodal form of PBL with distinct biological behavior, and it may also reflect the benefit of rapid and accurate diagnostic evaluation. The diagnosis was established using a complete diagnostic workup—including imaging, IHC, and FISH—with clonal relatedness confirmed by PCR and NGS, representing, to our knowledge, the first such comprehensive approach reported in the literature. Histologically, the axillary lymph node showed classic SLL features with CD20 and CD5 expression. In contrast, the retroperitoneal lymph node demonstrated a diffuse proliferation of large plasmablast- and immunoblast-like cells with high mitotic activity and necrosis. These cells expressed plasma cell markers CD38, CD138, and MUM1; had a high Ki-67 index; and lacked B-cell markers such as CD20 and PAX5, consistent with typical PBL [3,4,15]. The complete loss of PAX5 expression in the plasmablastic component represents an essential diagnostic criterion for plasmablastic lymphoma and supports its distinction from immunoblastic diffuse large B-cell lymphoma, in accordance with the WHO’s 5th edition classification [22]. Because PBL can overlap morphologically and immunophenotypically with other entities—including immunoblastic DLBCL, plasmablastic myeloma, ALK-positive large B-cell lymphoma, and HHV-8-associated DLBCL—these diagnoses were carefully excluded. In our case, ALK positivity and paraproteinemia were absent, and Burkitt lymphoma was ruled out based on characteristic morphology and immunophenotype, while the lack of BCL2 and BCL6 expression helped exclude DLBCL. Positivity for MYC and plasma cell markers supported the diagnosis of PBL [3,23].

Consistent with other reports, our patient tested negative for HIV, EBV, and HHV-8. Although HIV status is often negative or unreported in the literature, EBV positivity has been described by Khanna et al. and Martinez et al. ([2,7]; Table 1 and Table 2). These observations suggest that some cases of plasmablastic lymphoma arise independently of viral infection and instead represent clonal evolution of the original CLL/SLL clone rather than a de novo secondary lymphoma. NGS studies demonstrating identical immunoglobulin gene rearrangements in CLL and PBL components support a common clonal origin with acquisition of additional late genetic events. Central to this process is MYC deregulation, often through chromosomal rearrangements, driving high proliferative activity and transcriptional reprogramming toward terminal plasmablastic differentiation with loss of the mature B-cell phenotype, while the frequent absence of EBV in RT–PBL further supports a mechanism driven by somatic genetic alterations rather than viral oncogenesis.

Using FISH, we confirmed an MYC rearrangement, in line with the majority of previously reported cases ([2,6,9,17]). In contrast, Gasljevic et al. described a case without MYC rearrangement, highlighting potential diagnostic challenges, particularly as the antecedent disease was classical Hodgkin lymphoma rather than CLL/SLL ([11]; Table 2).

Confirming clonal relatedness through PCR and NGS was essential to establish this case as a true Richter transformation rather than two unrelated neoplasms ([2,6,12]). While Ramsay et al. and Chan et al. previously applied NGS, their analyses were limited to metachronous cases ([9,12]; Table 2). NGS is particularly valuable in synchronous transformations, where histopathological and immunophenotypic overlap can obscure disease evolution and where molecular confirmation is necessary to distinguish true transformation from the coexistence of two independent lymphoid neoplasms. In these instances, high-resolution genomic analysis provides definitive evidence of clonal relatedness, improves understanding of pathogenesis, and informs more tailored therapeutic strategies ([18]). Although additional synchronous transformation cases have been reported ([8,10,16,24,25]), they were excluded from our review due to a lack of clonality confirmation, limiting their interpretive value.

Therapeutic management of PBL remains challenging due to its intrinsic chemo-resistance, MYC rearrangements, plasmacytic phenotype, and often low CD20 expression, which reduces rituximab efficacy [12,17]. In most reported cases, patients received R-CHOP or CHOP-like regimens and succumbed within six months, with only two exceptions: one patient survived 24 months, and another remained stable at the time of publication ([7,13]; Table 2). A particularly unusual case, reported by Mazumder et al. but not included in our review due to unconfirmed clonality, involved spontaneous regression of a gingival mass following a biopsy [24]. However, the diagnostic workup was limited to histology and EBV positivity, without molecular or phenotypic profiling, leaving its classification uncertain. In contrast to most reviewed cases, particularly synchronous transformations, our patient was diagnosed approximately 24 months ago and remains clinically stable after completing eight cycles of chemotherapy. She was treated with a daratumumab-based regimen targeting CD38, a marker highly expressed in PBL, which led to rapid resolution of lymphadenopathy, hepatosplenomegaly, and systemic symptoms. The patient subsequently underwent ASCT and is maintained on daratumumab and ibrutinib. This multimodal approach appears effective in controlling both plasmablastic and residual B-cell components. These observations emphasize the importance of tailoring treatment to the plasmablastic phenotype, particularly through the use of CD38-targeted therapy, BTK inhibitors, and stem cell transplantation.

## 5. Conclusions

This report describes a rare synchronous transformation of CLL/SLL into PBL in an immunocompetent woman, with nodal disease and clonal relatedness confirmed by PCR and NGS. Early molecular confirmation allowed a prompt initiation of targeted multimodal therapy, resulting in a favorable outcome. These findings underscore the value of precise molecular diagnostics in synchronous transformations and the potential efficacy of CD38- and BTK-directed treatments in plasmablastic disease.

## Figures and Tables

**Table 1:** Overview of clinical and pathological features of reported transformation cases from CLL/SLL to PBL that were diagnosed simultaneously (under 6 months between diagnosis).

Reference	Gender and Age	HIV, EBV, HHV-8	Diagnostic Methods	Bone Marrow Involvement	FISH Analysis (MYC Rearrangements, BCL-2 and BCL-6)	Primary Localization	Therapy and Outcome
Our case	Female, 46 yrs	Negative	PET, LN biopsy (HE, IHC), flow cytometry, ISH, FISH, NGS	N/T	MYC rearrangements	Simultaneous diagnosis of CLL/SLL and PBL in axillary and retroperitoneal LN	Dara-CHOP (6×), autologous bone marrow transplantation, Dara-CHOP (4×), daratumumab, ibrutinib
[2]: 2 cases	Case 1: Female, elderly patient	Case 1: N/R	Case 1: BM biopsy (HE, IHC), flow cytometry, cytogenetic studies, FISH, transcriptome sequencing, PET	Consistent with CLL/SLL	Case 1: t (2; 3) in CLL and PBL, MYC-IGH fusion t (8; 14) and t (1; 6) in PBL	Case 1: 1 month, pleural fluid (cytology consistent with PBL)	Case 1: daratumumab, R-CHOP -patient died, surveillance N/A
[6]	Male, 61 yrs	Negative	Ultrasonography, axillary LN biopsy (HE, IHC), CT, PET, FISH	Both CLL/SLL and PBL	MYC gene rearrangement and translocation N/R	Simultaneous diagnosis of CLL/SLL and PBL in same left supraclavicular LN	Hyper-C-PAD -patient died several months after initial diagnosis
[7]: 3 cases	Case 1: Male, 70 yrs	Negative	Biopsies (HE, IHC), ISH, cytogenetic analysis, FISH	Case 1: CLL	Negative	Case 1: Simultaneous, mesenteric LN	Case 1: R-CHOP (2×) -patient died 4 months after diagnosis
[8]	Male, 42 yrs	HHV-8 positive Others N/R	CT scan, MRI, mediastinal biopsy and BM (HE, IHC), flow cytometry, cytogenetic analysis	Both CLL/SLL and PBL	N/R	Simultaneous diagnosis of CLL/SLL and PBL in bone marrow	High-dose steroids, radiation of mediastinal mass, CHOP, hyper-CVAD, intrathecal cytarabine -patient died in 3 months

Abbreviations: yrs—years; HIV—human immunodeficiency virus; EBV—Epstein–Barr virus; HHV-8—human herpesvirus 8; PET—positron emission tomography; LN—lymph node; HE—hematoxylin and eosin; IHC—immunohistochemistry; ISH—in situ hybridization; FISH—fluorescence in situ hybridization; NGS—next-generation sequencing; BM—bone marrow; MYC—MYC proto-oncogene; BCL2—B-cell lymphoma 2; BCL6—B-cell lymphoma 6; CLL—chronic lymphocytic leukemia; SLL—small lymphocytic lymphoma; PBL—plasmablastic lymphoma; Dara-CHOP—daratumumab plus cyclophosphamide, doxorubicin, vincristine, prednisone; R-CHOP—rituximab plus CHOP regimen; Hyper-C-PAD—hyperfractionated cyclophosphamide, cisplatin, doxorubicin, dexamethasone; Hyper-CVAD—hyperfractionated cyclophosphamide, vincristine, doxorubicin, dexamethasone; N/R—not reported.

**Table 2:** Overview of clinical and pathological features of reported transformation cases from CLL/SLL to PBL that were not diagnosed simultaneously (more than 6 months between diagnosis).

Reference	Gender and Age	HIV, EBV, HHV-8	Diagnostic Methods	Bone Marrow Involvement	FISH Analysis (MYC Rearrangements, BCL-2 and BCL-6)	Time and Localization	Therapy and Outcome
[2]: 2 cases	Case 2: Elderly patient	Case 2: EBV positive Others N/R	Case 2: BM biopsy (HE, IHC), flow cytometry, PCR, CT scan	CLL/SLL	Case 2: N/R	Case 2: Five years, plasmablast-like cells in pleural fluid	Case 2: R-CHOP (1x), DA-R-Velcade EPOCH * patient died, surveillance N/A
[9]	Male, 71 yrs	N/R	BM biopsy, CT scan, LN biopsy, FISH, NGS	CLL/SLL	MYC rearrangement	14 months, LN	CHOP (2x) -patient died after 4 months
[10]	Male, 53 yrs	HHV-8 positive Others N/R	LN and BM biopsies, genetic analysis, CT scan	First CLL, later PBL	N/R	7 years, bone marrow	COP regimen with added daratumumab from second cycle ** -patient died, surveillance N/A
[11]	Female, 74 yrs	EBV negative Others N/R	BM biopsy (HE, IHC), FISH, PCR, comparative genomic hybridization (CGH)	First CLL/SLL and later PBL	Without MYC rearrangements	7 years cHL with mixed cellularity in inguinal LN but PBL in bone marrow	No therapy *** -patient died 2–3 weeks after LN biopsy
[12]: 2 cases	Case 1: Male, 63 yrs Case 2: Male, 67 yrs	N/R	BM and mass biopsy, FISH, NGS	N/R	N/R	Case 1: 8 years, anal mass Case 2: 7 years, retroperitoneal LN	Salvage chemotherapy Case 1: patient died 2 weeks after PBL diagnosis Case 2: patient died 12 weeks after PBL diagnosis
[13]: 2 cases	Case 1: Male, 58 yrs Case 2: Male, 7 yrs	Case 1: Negative Case 2: Negative	LN and BM biopsy (HE, IHC), flow cytometry, cytogenetic studies, FISH, genetic studies	Case 1: CLL/SLL with plasmablasts Case 2: CLL/SLL	Case 1: CLL negative and PBL positive for MYC rearrangement Case 2: MYC rearrangement	Case 1: 2 years, ulcerated mass at the gastroesophageal junction and bone marrow. Case 2: Diagnosed with low-grade CD5+ LPD, after five years PBL left humerus	Case 1: N/A -patient died after 3 months Case 2: localized radiotherapy and chemotherapy -patient stable
[7]: 3 cases	Case 2: Male, 52 yrs Case 3: Female, 57 yrs	EBV positive in case 2 others N/R	Biopsies (HE, IHC), ISH, cytogenetic analysis, FISH	Both cases: CLL/SLL	Cases 2 and 3: N/R	Case 2: 85 months, subcutaneous tissue Case 3:47 months, mandibula	Case 2: R-CHOP (6x) -patient died 24 months after diagnosis of transformation Case 3: VAD (1x) and CHOP (3x) -patient died 6 months after diagnosis of transformation

* Breast carcinoma treated with chemotherapy; therapy for SLL/CLL—bendamustine and Rituxan. ** History of prostate cancer treated with hormone and radiotherapy; first therapy for CLL/SLL—fludarabine and cyclophosphamide; second therapy for CLL/SLL—fludarabine, cyclophosphamide and rituximab (6×) added ibrutinib. *** Early therapy—rituximab–chlorambucil with pegfilgrastim for CLL/SLL. Abbreviations: yrs—years; HIV—human immunodeficiency virus; EBV—Epstein–Barr virus; HHV-8—human herpesvirus 8; BM—bone marrow; HE—hematoxylin and eosin; IHC—immunohistochemistry; ISH—in situ hybridization; FISH—fluorescence in situ hybridization; NGS—next-generation sequencing; LN—lymph node; MYC—MYC proto-oncogene; BCL2—B-cell lymphoma 2; BCL6—B-cell lymphoma 6; cHL—classical Hodgkin lymphoma; PBL—plasmablastic lymphoma; CLL—chronic lymphocytic leukemia; SLL—small lymphocytic lymphoma; LPD—lymphoproliferative disorder; COP—cyclophosphamide, vincristine, prednisone; CHOP—cyclophosphamide, doxorubicin, vincristine, prednisone; R-CHOP—rituximab plus CHOP regimen; DA-R-Velcade EPOCH—daratumumab, rituximab plus Velcade, etoposide, prednisone, vincristine, cyclophosphamide, doxorubicin; VAD—vincristine, doxorubicin, dexamethasone; N/R—not reported.

## Data Availability

The data presented in this study are available on request from the corresponding author due to privacy.

## Supplementary Materials

The following supporting information can be downloaded at: https://www.mdpi.com/article/10.3390/diagnostics16050702/s1, Figure S1: Representative immunohistochemical and in situ hybridization findings in the plasmablastic lymphoma component. Immunohistochemistry shows complete absence of PAX5 expression in tumor cells, confirming loss of B-cell lineage markers. In situ hybridization for Epstein–Barr virus-encoded RNA (EBER) demonstrates absence of EBV-positive tumor cells.

## Abbreviations

RT	Richter’s transformation
CLL/SLL	Chronic lymphocytic leukemia/Small lymphocytic lymphoma
DLBCL	Diffuse large B-cell lymphoma
PBL	Plasmablastic lymphoma
HIV	Human immunodeficiency virus
EBV	Epstein–Barr virus
HHV-8	Human herpesvirus 8
SLL	Small lymphocytic lymphoma
CD	Cluster of differentiation
MUM1	Multiple myeloma oncogene 1
MYC	MYC proto-oncogene
Ki-67	Marker of proliferation
FISH	Fluorescent in situ hybridization
PCR	Polymerase chain reaction
IGH	Immunoglobulin heavy chain
IGK	Immunoglobulin kappa light chain
IGL	Immunoglobulin lambda light chain
NGS	Next-generation sequencing
PET	Positron emission tomography
HE	Hematoxylin and eosin
EMA	Epithelial membrane antigen
ALK1	Anaplastic lymphoma kinase 1
LANA1	Latency-associated nuclear antigen 1 (HHV8)
Dara-CHOP	Daratumumab + cyclophosphamide + doxorubicin + vincristine + prednisone
AHSCT	Autologous hematopoietic stem cell transplantation
BTK	Bruton tyrosine kinase
