# Supplementary material for: Plasmablastic Transformation of CLL/SLL: The Role of Early NGS Diagnosis and Targeted Multimodal Therapy
Source: Diagnostics (Basel). 2026 Feb 27;16(5):702. doi: 10.3390/diagnostics16050702 (PMC12984125; doi:10.3390/diagnostics16050702)
Supplement: Supplementary file 1 [file diagnostics-16-00702-s001.zip › diagnostics-4057535-supplementary.pdf]

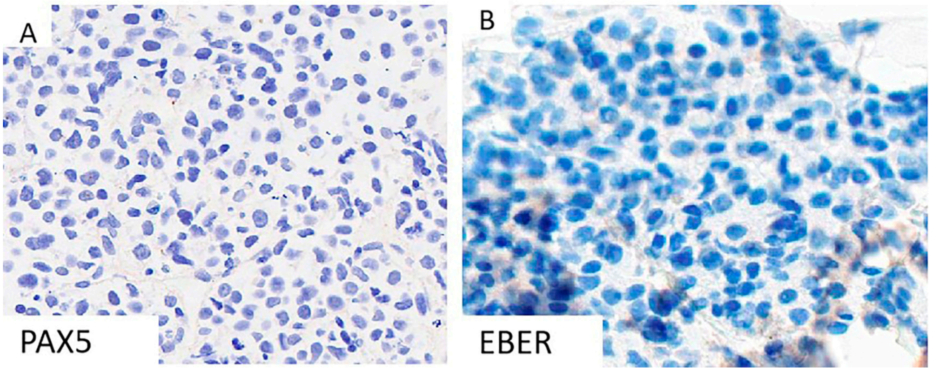

Supplementary Figure S1. Representative immunohistochemical and in situ hybridization findings in the plasmablastic lymphoma component. Immunohistochemistry shows complete absence of PAX5 expression in tumor cells, confirming loss of B-cell lineage markers. In situ hybridization for Epstein–Barr virus–encoded RNA (EBER) demonstrates absence of EBV-positive tumor cells.
